# Fatal Brazilian Spotted Fever Associated with Dogs and *Amblyomma aureolatum* Ticks, Brazil, 2013 

**DOI:** 10.3201/eid2512.191146

**Published:** 2019-12

**Authors:** Elisa S.M.M. Savani, Francisco B. Costa, Elisabete A. Silva, Ana C.F. Couto, Melanie Gutjahr, Juliana N.M.O. Alves, Fabiana C.P. Santos, Marcelo B. Labruna

**Affiliations:** Prefeitura de São Paulo, São Paulo, Brazil (E.S.M.M. Savani, E.A. Silva, A.C.F. Couto, M. Gutjahr, J.N.M.O. Alves);; Universidade de São Paulo, São Paulo (F.B. Costa, M.B. Labruna);; Instituto Adolfo Lutz, São Paulo (F.C.P. Santos)

**Keywords:** spotted fever, Brazilian spotted fever, *Rickettsia rickettsii*, *Amblyomma aureolatum*, tick, vector-borne infections, dogs, São Paulo, Brazil, rickettsia, bacteria, Rocky Mountain spotted fever

## Abstract

In São Paulo metropolitan area, Brazil, *Amblyomma aureolatum* ticks are the main vector of *Rickettsia rickettsii***,** which causes Brazilian spotted fever. In 2013, a boy in São Paulo died of Brazilian spotted fever associated with household dogs and *A. aureolatum* ticks. Prompt recognition and treatment of this illness might prevent deaths.

The bacterium *Rickettsia rickettsii* is the etiologic agent of Rocky Mountain spotted fever; in Brazil, this illness is called Brazilian spotted fever and is a national notifiable tickborne disease with fatality rates ≈50% (*1**,**2*). Since the 1920s, the vector of *R. rickettsii* in the southern São Paulo metropolitan area has been the *Amblyomma aureolatum* tick (*3**,**4*). In this area, free-roaming domestic dogs (major hosts of *A. aureolatum* ticks) are presumed to play a role in carrying *R. rickettsii–*infected ticks from forest fragments (*A. aureolatum* tick habitat) to household interiors (*4**,**5*). Dogs could thus be associated with the higher incidence of Brazilian spotted fever in women and children, who usually spend more time indoors, in close contact with dogs (*5*).

In November 2013, a 12-year-old boy died after 8 days of an acute febrile illness. He lived in the neighborhood of Sete Praias, near Atlantic forest remnants in the southern São Paulo metropolitan area. On day 3 of illness, he was admitted to the Nasf-Unifesp Hospital in the city of São Paulo with fever (temperature 39.5°C), headache, nausea, asthenia, and abdominal rash. The patient’s mother informed the physician that her son had been bitten by a tick on his nape ≈1 week before disease onset; the tick was removed and discarded. The boy was medicated with dipyrone and sent home. On day 6, the patient was returned to the hospital, unconscious, with jaundice and seizures. He was transferred to the intensive care unit; meningitis was suspected. The next day, his condition worsened, and when hematologic and biochemical examinations indicated thrombocytopenia and hepatic alterations, meningitis was ruled out. A blood serum sample was submitted for leptospirosis and spotted fever testing by serologic and molecular analysis, respectively. Results for leptospirosis were negative. The patient died on day 8 of illness. While the body was being prepared for the funeral, a tick was found attached behind the ear and was sent to the laboratory of the Prefeitura de São Paulo, where it was identified as an *A. aureolatum* unengorged female.

DNA extracted from the serum sample by use of PureLink Viral RNA/DNA Mini Kit (Invitrogen, https://www.thermofisher.com) was positive by Taqman real-time PCR for the genus *Rickettsia* (*6*). We therefore next performed 2 conventional PCRs, 1 targeting a 401-bp fragment of the rickettsial *gltA* gene (*7*) and the other targeting a 631-bp fragment of the rickettsial *ompA* gene (*8*). Both yielded amplicons that, after DNA sequencing, had sequences 100% identical to *R. rickettsii* (GenBank accession no. CP003305) by BLAST analyses (http://blast.ncbi.nlm.nih.gov/Blast.cgi).

Immediately after the patient’s death, the hospital notified the São Paulo Board of Health of this case, and we performed an epidemiologic investigation. In the patient’s household, we collected blood samples from 3 dogs and 11 cats, all adults, born and raised in the area, with free access to surrounding forests and the dwelling interior. Direct contact between the patient and his pets was reportedly common. Serum from the dogs and cats was tested for *R. rickettsii* IgG by immunofluorescence assay, as described (*3*). Seroreactivity was detected in the 3 dogs (endpoint titers 512, 2,048, and 4,096) and 3 of the cats (titers 64, 64, and 512).

During animal sampling, we collected 13 ticks from 1 dog and 1 tick from 1 cat; all ticks were *A. aureolatum* adults. These 14 ticks, plus the 1 from the patient’s body, were submitted for DNA extraction (*5*) and tested by the same 2 conventional PCRs. Two ticks (1 from the dog and the 1 from the patient) yielded *gltA* and *ompA* amplicons, which generated DNA sequences 100% identical to *R. rickettsii* (CP003305).

This fatal case of Brazilian spotted fever was epidemiologically associated with *A. aureolatum* ticks and domestic dogs. Because the patient had no recent history of traveling outside his neighborhood, we infer that he acquired the infection in his neighborhood, where *R. rickettsii* was circulating between ticks and his dogs. Although the *A. aureolatum* tick collected postmortem from the patient harbored *R. rickettsii,* we cannot be sure that this particular tick was the primary vector of the bacterium to the patient because the tick would certainly have been exposed to an infected blood meal during the last days of the patient’s life. We can, however, confirm that the patient was exposed in his neighborhood to *A. aureolatum* ticks, competent vectors of *R. rickettsii* (*5*). Because fed adult *A. aureolatum* ticks need only 10 minutes of attachment to transmit *R. rickettsii* to hosts (*5*), the likelihood of such transmission for this patient was high, considering his close contact with his pets. Had the physicians suspected Brazilian spotted fever when the boy was first admitted to the hospital on day 3 of febrile illness, treatment with appropriate antimicrobial drugs might have prevented his death (*9*).
